# Towards a future regulatory framework for chemicals in the European Union – Chemicals 2.0

**DOI:** 10.1016/j.yrtph.2023.105431

**Published:** 2023-08

**Authors:** Elisabet Berggren, Andrew P. Worth

**Affiliations:** European Commission, Joint Research Centre (JRC), Ispra, Italy

**Keywords:** Chemical safety assessment, Chemicals strategy for sustainability, Classification & labelling, CLP, New approach methodology (NAM), REACH, Regulatory toxicology, Roadmap, Risk management

## Abstract

The body of EU chemicals legislation has evolved since the 1960s, producing the largest knowledge base on chemicals worldwide. Like any evolving system, however, it has become increasingly diverse and complex, resulting in inefficiencies and potential inconsistencies. In the light of the EU Chemicals Strategy for Sustainability, it is therefore timely and reasonable to consider how aspects of the system could be simplified and streamlined, without losing the hard-earned benefits to human health and the environment.

In this commentary, we propose a conceptual framework that could be the basis of Chemicals 2.0 – a future safety assessment and management approach that is based on the application of New Approach Methodologies (NAMs), mechanistic reasoning and cost-benefit considerations. Chemicals 2.0 is designed to be a more efficient and more effective approach for assessing chemicals, and to comply with the EU goal to completely replace animal testing, in line with Directive 2010/63/EU.

We propose five design criteria for Chemicals 2.0 to define what the future system should achieve. The approach is centered on a classification matrix in which NAMs for toxicodynamics and toxicokinetics are used to classify chemicals according to their level of concern. An important principle is the need to ensure an equivalent, or higher, protection level.

## Highlights

• There is a need to assess and manage chemicals more efficiently and effectively.
• Chemicals 2.0 is a vision for a future safety assessment and management framework.
• We propose five design criteria for Chemicals 2.0
• NAM-based toxicodynamic and toxicokinetic data classify chemicals in concern levels.
• A multi-stakeholder collaboration would develop the approach and build confidence.

## 1 Introduction

There is a pressing need to better assess and manage the risks of chemicals, to enable a sustainable progress towards the use of less hazardous chemicals (EEA, 2023). However, a number of challenges need to be overcome.

A major challenge is the sheer scale of the assessment exercise. The European Environment Agency (EEA, 2019) has estimated that robust information exists for only about 0.5% of the chemicals on the market, while 10% are fairly well characterised and another 20% have very limited hazard and exposure information available. This leaves approximately 70% of the chemicals on the EU market not assessed for their safe use. This large percentage, albeit uncertain, is due to the fact that most manufactured chemicals are not subject to any legal act requiring information to ensure safety.

In the European Union (EU), the Registration, Evaluation, Authorisation and Restriction of Chemicals (REACH) regulation (EU, 2006) is the primary cross-cutting legislation aimed at managing the risks of industrial chemicals. REACH obliges companies to generate information on the intrinsic properties of chemicals that are manufactured in, or imported into, the EU. This information is used to classify chemicals on the basis of their hazardous properties by means of the Classification, Labelling and Packaging (CLP) regulation (EU, 2008), which in itself does not include any information requirements, but implements, in the EU, the classification criteria for hazardous chemicals of the United Nations Globally Harmonised System (UN, 2021). REACH and CLP are complemented by sectorial legislation dealing with chemicals in products, waste and environmental media. Under REACH, information requirements are proportionate to marketed tonnage levels. No information is requested below 1 tonne per year, and the information required in the 1–10 tonnes band is sufficient neither for a Chemical Safety Assessment nor for classification under CLP. At the time of writing, around 14,000 unique substances are subject to REACH (ECHA, 2023), while smaller numbers of chemicals are covered by sectorial legislation, such as plant protection products, biocides and cosmetics. In other words, the body of EU chemicals legislation, in spite of its complexity including more than 40 legal acts (EC, 2019), is not designed to address all of the chemicals on the EU market.

A second major challenge concerns the efficiency with which chemicals are assessed and managed. It has been argued (EEB, 2022) that the current REACH procedures are inefficient, with harmonised classifications taking an average of 19 years and five months, REACH restrictions 19 years and three months, and the phasing out of chemicals of very high concern under REACH authorisation taking 22 years and 11 months. The chemicals that are most efficiently assessed are the pesticides (EU, 2011) and biocides (EU, 2012). Under these pieces of sectorial legislation there are well defined information requirements necessary to fulfil for authorisation to use the products including these chemicals on the EU market, and the assessment is in both cases made by expert groups. The information requirements also provided the necessary information for classification under CLP, but the procedure is extremely resource intensive.

The Chemicals Strategy for Sustainability (CSS), adopted by the Commission in 2020 (EC, 2020), aims to improve this situation. The Strategy envisages that chemicals should be produced and used safely and sustainably by 2030. The CSS includes 85 actions to avoid the negative impacts of chemicals on human health and environment, while fully exploiting their benefits for the economy and society. Among the actions of the CSS are targeted revisions of REACH and the CLP regulations, along with several other sectorial pieces of legislation containing provisions on chemicals. These revisions, while extensive and ambitious, will only go so far in the short time frame to prepare a legislative proposal. This means it is timely to consider what additional steps need to be taken to ensure the longer-term goals of the CSS are addressed. Within the context of the REACH information requirements, the Commission has committed to develop a roadmap that will increasingly rely on emerging technologies, so-called New Approach Methodologies (NAMs) (ECHA, 2016), with the eventual aim of replacing animal testing in the regulatory assessment of chemicals. Similarly, EFSA has developed roadmaps to adopt NAMs and phase out animal testing in the implementation of the food and feed legislation (EFSA, 2020; Cattaneo et al., 2023).

In this commentary, we provide some ideas to frame and stimulate discussions on how the chemicals legislation might evolve to increase the effectiveness and efficiency of chemical assessment process, while also phasing out animal testing. The focus is on how chemicals are classified according to their hazardous properties and the implications of classification for further assessment and/or risk management.

## 2 Problems experienced when introducing NAMs under the REACH information requirements

The CLP classification criteria for systemic toxicity and environmental endpoints are largely based on traditional animal studies, and the corresponding information requirements under REACH refer to international accepted guidelines in the Organisation for Economic Co-operation and Development (OECD) Test Guidelines (TGs) programme (https://www.oecd.org/chemicalsafety/testing/oecd-guidelines-testing-chemicals-related-documents.htm) to obtain the relevant data to fulfil the criteria. A survey carried out by the Commission's Joint Research Centre (JRC) revealed that NAM-based strategies are already used by manufacturers and downstream users in internal evaluations of chemicals, but the NAMs are not considered sufficiently validated or standardised to include in legislation, such as the REACH regulation. Moreover, the international validation and acceptance process is time-consuming (up to 10 years), with the result that the number of NAMs introduced into a legal instrument is very limited. The rate of adoption of NAM-based TGs is increasing, but still quite low, with approximately 1–4 TGs adopted per year. So far, these new TGs have predominantly been in the areas of skin and eye irritation, skin sensitisation, endocrine activity and genotoxicity, as opposed to the systemic toxicities (V. Zuang, personal communication).

In addition, there is a general perception that the NAMs are “less safe” and introduce a higher level of uncertainty, at least when used as standalone methods. For this reason, NAMs are generally accepted in screening level assessments, which may then trigger higher tier animal testing. As a consequence, the introduction of NAMs under the REACH information requirements is more likely to lead to additional animal testing, and a costly and lengthy assessment, rather than to a reduction of animal testing and a more efficient assessment. An additional issue is the applicability domain of NAMs, often considered to be the fraction of substances that can be assessed when compared with animal testing (i.e. excluding substances that are difficult to test in a cell-based system). However, all methods, animal or non-animal, have limitations in their applicability depending on their physicochemical properties (Netzeva et al., 2005). It is therefore important to identify and compare these limitations to gain acceptance of NAMs and also highlight where individual methods provide added value.

Systemic health effects include a range of biological (key) events leading to an adverse outcome or disease. Since a 1-to-1 replacement of an animal assay is not possible with standalone NAMs, several methods need to be integrated to cover the relevant modes of action (OECD, 2017). This is challenging in itself, but even though an integrated approach could in principle cover the relevant biology, the results obtained would not provide legal certainty for CLP classification. This is because the criteria are based on observable effects in intact animals, traditionally considered as the “gold standard” in regulatory testing. This indicates that there is not only a need to find NAM solutions to fit the data requirements, but also the legal requirements must be adapted to the new methodologies.

## 3 “Chemicals 2.0” – a long-term objective for chemical safety assessment

The CSS (EC, 2020) acknowledges that some hazard properties are of higher concern than others, and in particular makes reference to so-called “chemicals of concern” that have a chronic effect for human health or the environment. One of the goals of the CSS is to ensure that chemicals of concern are minimised and substituted as far as possible. In practice, this means that new CLP classes are needed to identify such chemicals and form the basis of the generic approach to risk management.

There are two major challenges in introducing a new hazard class. First there must exist standardised methodologies to detect the hazard, such as OECD TGs. Second, the resulting risk management should lead to a higher degree of safety.

Additional hazard classes bring costs in terms of the time needed to restrict the use of hazardous substances, the testing requirements for manufacturers, and the number of tests (be they animal-based or NAM-based). It is therefore important and complex to evaluate the added value for safety when introducing additional hazard classes under GHS and CLP. The current systemic health hazards are already overlapping since common underlying biological mechanisms lead to different endpoints (Madia et al., 2021).

We need to achieve a better appreciation for what is good enough to protect human health and the environment. A first obvious goal would be to try to cover a larger proportion of the chemicals on the market, especially the substances that remain unassessed today, while also being more confident that the chemicals used for replacement are of low hazard. Following on from that, prioritisation of chemicals for further testing and assessment would be based on concern rather than the market volume, as is currently the case under REACH.

## 4 Design specifications of a future regulatory system

Let's stop patchworking the system and re-think its design. As an aspirational thought starter, we propose for the following criteria. The future chemicals safety system should:

• be applicable to all substances on the market
• provide an equivalent level of protection (same risk management decisions) as under current legislation
• maintain current CLP classifications
• manage risks based on the use of NAMs for both toxicodynamic and toxicokinetic properties
• provide regulatory certainty (there is a level playing field in which authorities and companies follow agreed methodology) and guide innovation

## 5 Principle of equivalent protection

The principle of equivalent protection means that the same risk management decisions would be reached by using NAMs instead of animals. By keeping the current classification conclusions, the current hazard-based risk management of chemicals is not changed and there is no lowering of protection levels. This makes sense as classifications are based on knowledge and experience that should not be lost, even though the extent and standard of evidence might vary considerably between different chemicals. In other words, existing classifications should be considered as sound, unless new evidence indicates otherwise. At the same time, a higher level of overall protection derives from additional NAM-based classification conclusions for a broader range of chemicals.

## 6 Developing a new classification scheme based on toxicodynamic and toxicokinetic properties

The new classification scheme is designed to lead to the same levels of protection, without necessarily aiming to predict adverse effects in animal studies. The success of the new scheme will be judged on its ability to trigger the same risk management decisions for currently assessed chemicals, while also informing on the safe use or risk assessment needs of the large number of unassessed chemicals.

**Fig. 1:**
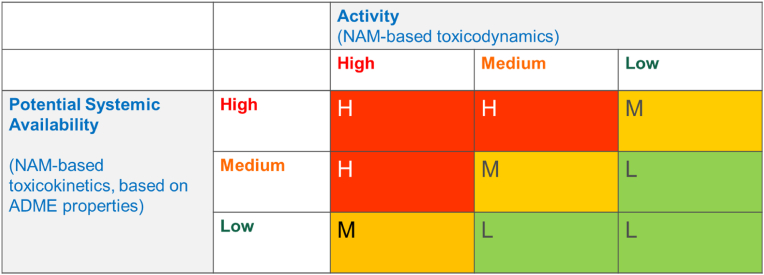
A new classification scheme for chemicals based on three levels of concern (High, Medium and Low).

As illustrated in Fig. 1, the new classification scheme will rank chemicals into three levels of concern (high, medium and low) based on toxicodynamic (TD) and toxicokinetic (TK) considerations. The TD and TK properties are independent of the external exposure scenario. This avoids reliance on external exposure information, which is often not available or unreliable. These properties will also be assessed entirely on the basis of NAMs, thereby avoiding animal testing.

The TD assessment will take into account not only the potency but also whether the property is considered to be of high concern (e.g. carcinogenicity).

The TK assessment will be based on NAMs for Absorption, Distribution, Metabolism and Elimination (ADME) resulting in a TK ranking that reflects the potential systemic availability of the chemical. In this context, potential systemic availability is taken to depend not only on metabolism and persistence, but also bioaccumulation over time.

The proposed classification scheme is similar to a recently proposed approach to the classification of carcinogenicity (Doe et al., 2022). In this respect, the three-level classification system, in which TK and TD considerations are considered independently, partially addresses several critiques of the current binary classification system (Boobis et al., 2016; Cohen et al., 2019; Doe et al., 2019).

## 7 Consequences for risk management

Chemicals ending up as high concern (in the red zone) would be banned or severely restricted. To use these substances, authorisation for specific uses should be required. The medium concern chemicals (orange zone) are the ones that would be possible to use with restrictions, following chemical-specific risk assessments for certain uses. The low concern chemicals (green zone) can be used without restriction, enabling the innovation of safe and sustainable materials.

## 8 Phasing in the new classification scheme

The transition to a new classification system should be gradual and build on the current one. The experience already invested in classifications in the past is preserved in the new system. The current scheme can exist in parallel with the new one until the latter is proven to provide enough protection. All stakeholders get the opportunity to gain confidence and where needed revise the new criteria, before the current system, still to a certain extent based on animal testing, is phased out. During this period, methodologies supporting the new classification scheme can be the basis for revision of criteria and information requirements in the current legislation, providing a degree of continuity between the two systems and progressively reducing animal testing.

## 9 Design of testing strategies

The selection of NAMs to include in the assessment would be based on multiple considerations, including level of validation and standardisation, deployability, mode of action reasoning, and cost-effectiveness (added value in the assessment). A first step would serve to judge whether the substance might be of concern or not. It is important to note that today the chemicals considered as low concern are often completely uncharacterised, so even a modest increase in knowledge should create better confidence in the selection of less hazardous chemicals.

The new scheme based on NAMs should also be proportionate, only adding test requirements when there is a clear added value to the overall assessment, with suitable methods being specified in guidance on testing strategies. This implies the need to apply methodology for cost-effectiveness assessment into the testing regime (Norlen et al., 2014). For example, new concerns are more efficiently addressed by adding for example a battery of NAMs for developmental neurotoxicity, but selecting only the individual methods providing an additional value to the already applied NAM battery.

## 10 Validation and standardisation of NAMs

Compared to the current, highly resource-intensive, approach to validation and regulatory acceptance of NAMs, the future system needs multiple pathways to acceptance, to encourage innovation and rapid uptake of new methods in the chemical safety assessment. This means that the validation process should be fit-for-purpose, and consideration given to the appropriate level of acceptance (EU-specific agency, EU-wide or international).

NAMs used for classification and labelling (identifying chemicals of medium and high concern) should be well defined, independently validated and standardised at EU or international level, to create the basis for legal certainty.

NAMs used for risk assessment (establishing safe levels for chemicals of medium concern) can be more flexible, allowing companies to develop their own bespoke solutions including non-standard methods as long as EU-harmonised guidance on performance characteristics is developed and respected. This can be considered a means of promoting innovation while gaining experience and confidence in new NAMs.

The need to gain efficiencies in the validation and acceptance of NAMs is a topic of international debate, with various “confidence frameworks” being proposed in the literature (Parish et al., 2020; Patterson et al., 2021; van der Zalm et al., 2022).

## 11 Conclusions

The body of EU chemicals legislation has evolved since the 1960s, producing the largest knowledge base on chemicals worldwide. Like any evolving system, however, it has become increasingly diverse and complex, resulting in inefficiencies and potential inconsistencies. In the light of the Chemicals Strategy for Sustainability, it is therefore timely and reasonable to consider how aspects of the system could be simplified and streamlined, without losing the hard-earned benefits to human health and the environment.

In this commentary, we have outlined a conceptual framework that could be the basis of Chemicals 2.0 – a future safety assessment and management approach that is based on the application of NAMs, mechanistic reasoning and cost-benefit considerations. Chemicals 2.0 is designed to be a more efficient and more effective approach for assessing chemicals, complying with the EU goal to completely replace animal testing, as stipulated in Directive 2010/63/EU.

In the transition towards Chemicals 2.0., it will first be necessary to design the overall testing strategy, and to calibrate the decision points to ensure an equivalent level of protection for those substances that are already assessed and risk managed. In a second stage, the calibrated system will be used to assess the wider universe of currently unassessed chemicals. Some of these will be prioritised for further testing and assessment, potentially leading to new risk management measures. In other cases, the additional information will inform monitoring campaigns to establish exposure patterns for safe use. It is suggested that the new approach is developed and tested in parallel with the current system as a means of gaining confidence. NAM solutions envisaged for Chemicals 2.0 could also be introduced to partially fulfil information requirements and classification criteria under current legislation, to ensure a stepwise transition towards the new system. If this multi-stakeholder endeavour is successful, there will come a point in time when the new system becomes mainstream while the current system is superseded. Building on the evolution metaphor (Landsiedel et al., 2022), it is time to tend the “Regulatory Garden”.

## Credit author statement

Elisabet Berrgren: Conceptualization, Writing, Editing.

Andrew Worth: Conceptualization, Writing, Editing.

## Disclaimer

This article reflects the views of the authors and does not necessarily reflect those of the European Commission.

## Funding body information

This work was fully funded by the European Commission's Joint Research Centre.

## Declaration of competing interest

The authors declare that they have no known competing financial interests or personal relationships that could have appeared to influence the work reported in this paper.

## Data Availability

No data was used for the research described in the article.
